# Capsaicin Is a Negative Allosteric Modulator of the 5-HT_3_ Receptor

**DOI:** 10.3389/fphar.2020.01274

**Published:** 2020-08-31

**Authors:** Eslam El Nebrisi, Tatiana Prytkova, Dietrich Ernst Lorke, Luke Howarth, Asma Hassan Alzaabi, Keun-Hang Susan Yang, Frank C. Howarth, Murat Oz

**Affiliations:** ^1^Department of Pharmacology, College of Medicine and Health Sciences, UAE University, Al Ain, United Arab Emirates; ^2^Department of Pharmacology, Dubai Medical College, Dubai Medical University, Dubai, United Arab Emirates; ^3^MolOptima Biotech Inc., Santa Margarita, CA, United States; ^4^Department of Anatomy and Cellular Biology, Khalifa University, Abu Dhabi, United Arab Emirates; ^5^Department of Cellular Biology and Pharmacology, Herbert Wertheim College of Medicine, Florida International University, Miami, FL, United States; ^6^Department of Biological Sciences, Schmid College of Science and Technology, Chapman University, Orange, CA, United States; ^7^Department of Physiology, College of Medicine and Health Sciences, UAE University, Al Ain, United Arab Emirates; ^8^Department of Pharmacology and Therapeutics, Faculty of Pharmacy, Kuwait University, Safat, Kuwait

**Keywords:** capsaicin, 5-HT_3_ receptor, Xenopus oocytes, HEK-293 cells, serotonin, allosteric modulator, docking

## Abstract

In this study, effects of capsaicin, an active ingredient of the capsicum plant, were investigated on human 5-hydroxytryptamine type 3 (5-HT_3_) receptors. Capsaicin reversibly inhibited serotonin (5-HT)-induced currents recorded by two-electrode voltage clamp method in *Xenopus* oocytes. The inhibition was time- and concentration-dependent with an IC_50_ = 62 μM. The effect of capsaicin was not altered in the presence of capsazepine, and by intracellular BAPTA injections or trans-membrane potential changes. In radio-ligand binding studies, capsaicin did not change the specific binding of the 5-HT_3_ antagonist [^3^H]GR65630, indicating that it is a noncompetitive inhibitor of 5-HT_3_ receptor. In HEK-293 cells, capsaicin inhibited 5-HT_3_ receptor induced aequorin luminescence with an IC_50_ of 54 µM and inhibition was not reversed by increasing concentrations of 5-HT. In conclusion, the results indicate that capsaicin acts as a negative allosteric modulator of human 5-HT_3_ receptors.

## Introduction

Capsaicin, a unique alkaloid extracted from Chili peppers of Capsicum family, is responsible for the hot pungent taste of this plant. Capsaicin together with dihydrocapsaicin constitute nearly 90% of the capsaicinoid alkaloids found in chili pepper (O’Neill et al., 2012). In recent years, therapeutic effects of capsaicin have been gaining increasing interest in various fields of medicine ranging from analgesia, anti-inflammation, and obesity to treatment of cancer (Sharma et al., 2013; Srinivasan, 2016; Patowary et al., 2017; Zhang et al., 2020).

In earlier studies, it has been well established that capsaicin causes its pain-relieving effect by activating and desensitizing the capsaicin receptor, which is known as “Transient receptor potential cation channel, subfamily V, member 1” (TRPV1). TRPV1 is a non-selective, Ca^2+^ permeable cation channel activated by protons, noxious heat, endogenous lipids, and exogenous ligands, such as resiniferatoxin and capsaicin (Lumpkin and Caterina, 2007; O’Neill et al., 2012). Although, the activation of TRPV1 is considered to be an important mechanism, the exact nature of the widely ranging biological actions of capsaicin is currently unknown.

The serotonin type three (5-HT_3_) receptor is a member of the cys-loop family of ligand-gated ion channels. Fast depolarizing synaptic actions of 5-HT are mediated by 5-HT_3_ receptors in the central and peripheral nervous systems (Thompson and Lummis, 2006). Currently, 5-HT_3_ receptor antagonists are used in clinics for the treatment of chemotherapy-induced nausea, vomiting, and irritable bowel syndrome (Thompson and Lummis, 2007; Binienda et al., 2018). In recent years, there has been renewed interest in exploring the therapeutical potential of 5-HT_3_ receptor modulators in various neuropsychiatric disorders such as schizophrenia, depression, anxiety, and drug abuse (Fakhfouri et al., 2019; Juza et al., 2020). In the present study, using electrophysiological and biochemical methods, we have investigated the effect of capsaicin on the functional properties of human 5-HT_3_ receptors expressed in *Xenopus* oocytes and HEK-219 cells.

## Materials and Methods

Mature female *Xenopus laevis* frogs were obtained from *Xenopus* leavis I, Ann Arbor, MI, USA. Experiments and methods used in this study were in accordance with the Guide for the Care and Use of Laboratory Animals of the National Institutes of Health (Bethesda, MD, USA) and our protocol (A9/08) was approved by the Institutional Animal Care and Use Committee at the College of Medicine and Health Sciences, United Arab Emirates University. Clusters of oocytes were removed surgically under benzocaine (0.03% w/v; Sigma, St.Louis, MO) anesthesia. Individually dissected oocytes were stored for 2 to 8 days in modified Barth’s solution (MBS) containing (in mM): NaCl 88; KCl 1; NaHCO_3_ 2.4; CaCl_2_; 2; MgSO_4_ 0.8; HEPES 10 (pH 7.5), supplemented with sodium pyruvate 2 mM, penicillin 10,000 IU/L, streptomycin 10 mg/L, gentamicin 50 mg/L, and theophylline 0.5 mM. Human 5-HT_3A_ receptor cRNA (3 ng in 50 nl) was injected into each oocyte as described before (Ashoor et al., 2013). In co-expression of subunit combinations, cDNAs for 5-HT_3A_ and 5-HT_3B_ subunits, were mixed in ratios of 1:1 (or 1:2), respectively. Following day, oocytes were placed in a 0.2 ml recording chamber and superfused at a constant rate of 3 to 5 ml/min. The bathing solution consisted of: 95 mM NaCl; 2 mM KCl; 2 mM CaCl_2_; and 5 mM HEPES 5 (pH 7.5). The oocytes were impaled with two standard glass microelectrodes filled with a 3 M KCl (1–3 MΩ) and voltage clamped at a holding potential of −70 mV using GeneClamp-500B amplifier (Axon Instruments Inc., Burlingame, CA). Current responses were digitized by A/D converter and analyzed using pClamp 10.4 (Molecular Devices-Axon Instruments, San Jose, CA USA) or Origin™ (Originlab Corp. Northampton, MA, USA), run on an IBM/PC. Compounds were applied by addition to the superfusate. Capsaicin ((*E*)-N-[(4-Hydroxy-3-methoxyphenyl)methyl]-8-methyl-6-nonenamide, ≥98%; Cat. No. 0462), capsazepine (≥99%; Cat. No. 0464), BAPTA (≥95%; Cat. No. 2786/100), 5-HT, 2-methyl-5HT, and MDL72222 (Tropanyl 3, 5-dichlorobenzoate; ≥99; Cat. No. 0640) were purchased from Tocris Cookson (St. Louis, MO). Dihydrocapsaicin (98%; Cat. No. 03813), vanillin (99% Cat. No. V1104), and all chemicals used in preparing the solutions were provided by Sigma-Aldrich (St. Louis, MO, USA). Procedures for the injections of BAPTA (50 nl, 100 mM) were performed as described previously (Oz et al., 1998). Injections were performed 10 min prior to recordings using oil-driven ultra microsyringe pumps (Micro4; WPI, Inc. Sarasota, FL, USA). Stock solutions of capsaicin were prepared in DMSO. Vehicle (DMSO) alone did not affect 5-HT_3_ receptor function when added at concentrations as high as 0.3% (v/v), a concentration twice above the most concentrated application of the agents used.

### Synthesis of cRNA

The cDNA clones of the human 5-HT_3A_ and 5-HT_3B_ subunits were provided by OriGen Inc. (Rockville, MD). Complementary RNAs (cRNAs) were synthesized *in vitro* using a mMessage mMachine RNA transcription kit (Ambion Inc., Austin, TX). The quality and size of synthesized cRNAs were confirmed by denatured RNA agarose gels.

### Radioligand Binding Studies

Oocytes were injected with 10 ng human 5-HT_3_ cRNA, and functional expression of the receptors was assessed by electrophysiology on day three. Isolation of oocyte membranes was carried out by modification of a method described earlier (Oz et al., 2004). Briefly, oocytes were suspended (20 μl/oocyte) in a homogenization buffer (HB) containing HEPES 10 mM, EDTA 1 mM, 0.02% NaN_3_, 50 μg/mL bacitracin, and 0.1 mM PMSF (pH 7.4) at 4°C on ice and homogenized using a motorized Teflon homogenizer (six strokes, 15 s each at high speed). The homogenate was centrifuged for 10 min at 800 g. The supernatant was collected, and the pellet was resuspended in HB and re-centrifuged at 800*g* for 10 min. Supernatants were then combined and centrifuged for 1 h at 36,000*g*. The membrane pellet was resuspended in HB at the final protein concentration of 0.5 to 0.7 mg/ml and used for the binding studies.

Binding assays were performed in 500 μl of 10 mM HEPES (pH 7.4) containing 50 μl of oocyte preparation and 1 nM [^3^H]GR65630 (Perkin-Elmer, Inc. Waltham, MA, USA). Nonspecific binding was determined using 100 μM MDL72222. Oocyte membranes were incubated with [^3^H]GR65630 in the absence and presence of capsaicin at 4°C for 1 h before the bound radioligand was separated by rapid filtration onto GF/B filters pre-soaked in 0.3% polyethylenemine. Filters were then washed with two 5-mL washes of ice-cold HEPES buffer and left in 3 mL of scintillation fluid for at least 4 h before scintillation counting was conducted to determine amounts of membrane-bound radioligand.

### Aequorin Luminescence Assay

Luminescence experiments were performed according to methods and protocols described earlier (Walstab et al., 2007), with some modifications. Human embryonic kidney (HEK 293) cells stably expressing apoaequorin (HEK293-AEQ17 cells; Button and Brownstein, 1993) were cultured as described previously for HEK-293/EM4 cells (Oz et al., 2010). Cells were seeded in 25-cm^2^ cell culture flasks in Dulbecco’s modified Eagle’s medium (DMEM)/Ham’s F12 (1:1) + 10% fetal bovine serum to obtain a cell density of 50% to 70%, and the following day, transiently transfected with cDNA encoding human 5-HT_3A_ receptor (3 µg) using Lipofectamine 2000 reagent (Thermo Fisher Scientific-Invitrogen, Waltham, MA) according to the manufacturer’s instructions. Two days after transfection, cells were harvested by centrifugation and resuspended in 0.5 ml (25-cm^2^ flask) DMEM/Ham’s F12 (1:1) + 0.1% bovine serum albumin. The cell suspension was incubated with 10 μM Coelenterazine *h* (Thermo Fisher Scientific-Invitrogen, Waltham, MA, USA) for three hours at room temperature in the dark. After loading, cells were harvested by centrifugation and resuspended in assay buffer containing 150 mM NaCl, 1.8 mM CaCl_2_, 5.4 mM KCl, 10 mM Hepes, and 20 mM D-glucose at pH 7.4 at the approximate cell density of 3 to 5 × 10^6^ cells/ml. Cell suspension (60 μl) was preincubated with 20 µl capsaicin in a 96-well plate for 10 min. at room temperature and activated by 20 μl of 10 μM 5-HT injection. Luminescence was measured using a Luminoskan (Thermo Fisher Scientific, Waltham, MA, USA) equipped with an injector and recorded at a sampling rate of 2 Hz for up to 60 s. At the end of the experiments, cells were lysed with Triton X-100 0.1% (v/v) and CaCl_2_ 50 mM, and aequorin luminescence was recorded to obtain the maximum Ca^2+^ response. Each capsaicin concentration was measured in quadruplicates in two experiments. Data were exported to software Origin™ 8.5 (Originlab Corp. Northampton, MA, USA). Peak values in relative light unit (RLU) for 5-HT responses were obtained by subtraction of baseline luminescence from the agonist-induced peak luminescence and normalizing to maximal Ca^2+^ response.

### Data Analysis

For the nonlinear curve fitting and regression fits of the dose-response curves and radio-ligand binding data, the computer software Origin™ 8.5 (Originlab Corp. Northampton, MA, USA) was used. In functional assays, average values were calculated as mean ± standard error means (S.E.M.). Statistical significance was analyzed using ANOVA or Student’s *t* test and *post hoc* Bonferroni test was used following ANOVA. Concentration-response curves were obtained by fitting the data to the logistic equation,

$$y=\{(E_{\max}-E_{\min})/(1+{\left[EC_{50}\right]}^{n})\}+E_{\min},$$

where x and y are concentration and response, respectively, E_max_ is the maximal response, E_min_ is the minimal response, EC_50_ is the half-maximal concentration, and n is the slope factor.

### Docking Studies

Docking calculations were performed on 5HT_3_ receptor (Protein Data Bank ID code 4PIR (Hassaine et al., 2014)). Docking of compounds capsaicin, dihydrocapsaicin, vanillin, and capsazepine to structural model was made by Autodock Vina program (Trott and Olson, 2010), results were verified using Gold docking software, which is part of CSD Discovery suite from Cambridge Crystallographic Data Center (Groom et al., 2016). Ligand files were downloaded from PubChem structural database (Kim et al., 2018). Ligand and receptor files were prepared using m Autodock Tools (ADT) (Morris et al., 2009). Polar hydrogens, united atoms Kollman charges and solvation parameters were identified, files were saved in PDBQT format. Affinity grid maps of 30 Å × 30 Å × 30 Å with spacing 0.375 Å were added. Grid center was designated x, y, z dimensions: 139.00, 219.00 and 273.00. These coordinates correspond to allosteric binding site of 5HT_3_ receptor for ginger compounds identified in an earlier study (Lohning et al., 2016) on human 5HT_3_ receptor. Docking calculations were performed using the Lamarckian genetic algorithm (LGA) (Morris et al., 1998). During the docking procedure, both the protein and ligands were considered as rigid. The poses with lowest binding free energy were aligned with receptor for further analysis of interactions. Binding poses were verified by Gold docking program. In GOLD docking was prepared using the Hermes program and wizard for docking with default parameters such as population size (100); selection- pressure (1.1); number of operations (10,000); number of islands (1); niche size (2); and operator weights for migrate (0), mutate (100), and crossover (100). The active site with a 10 Å radius sphere was defined by selecting an active site residue of protein. Default Genetic Algorithm settings were used for all calculations and a set of 10 solutions were saved for each ligand. GOLD was used by a GoldScore fitness function.

## Results

In initial experiments, fast inward currents activated by 5-HT (1 μM) or 2-methyl-5-HT (10 μM) were completely inhibited by 0.5 µM MDL72222, a specific 5-HT_3_ receptor antagonist, indicating that functional 5-HT_3_ receptors are expressed in *Xenopus* oocytes (n = 7). Capsaicin (100 µM for 1 min) alone did not induce current responses in oocytes expressing 5-HT_3_ receptors in the absence (n = 5) and presence of 0.5 µM MDL72222 (n = 5).

**Figure 1:**
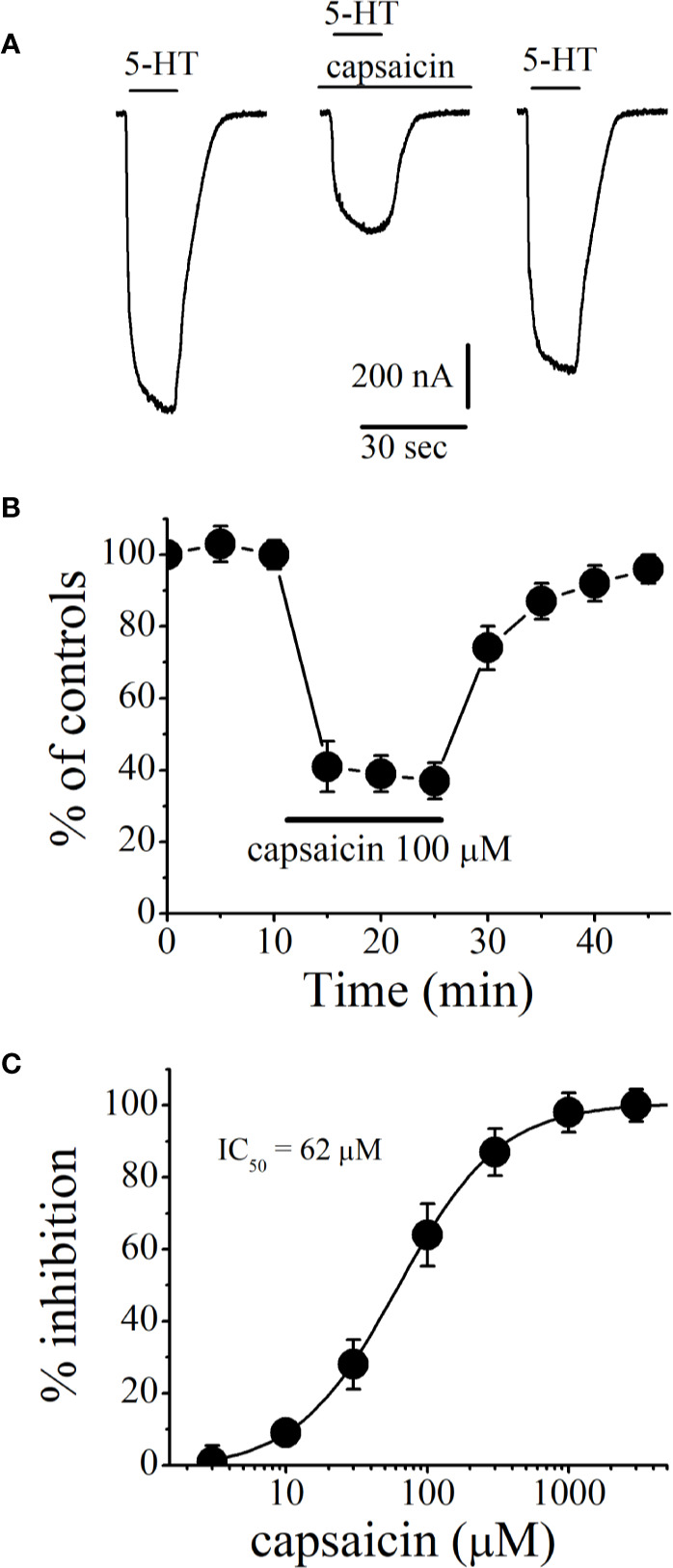
Effects of capsaicin on the function of human 5-HT_3_ receptors expressed in *Xenopus* oocytes. **(A)** Representative traces of currents activated by 5-HT (1 µM; on the *left*), coapplication of 5-HT and 100 µM capsaicin after 10 min capsaicin pre-application (*middle*), 20 min wash-out (*right*). **(B)** Effect of capsaicin application on the normalized amplitudes of currents activated by 5-HT (1 µM) at 5 min intervals. Current amplitudes were normalized to first agonist application in each experiment. Solid bar represents application time for capsaicin (100 µM). Data points represent means ± S.E.M. of 7–8 cells. **(C)** Capsaicin inhibits the function of 5-HT_3_ receptor in concentration-dependent manner. For all concentrations used, capsaicin was applied for 10 min. Data points represent mean ± S.E.M. (n = 6–8).

**Figure 1A** shows the recordings of currents activated by 5-HT (1 μM) in control (on the *left*), after 10 min capsaicin (100 µM) application (in the *middle*), and after 20 min of washout (on the *right*). **Figure 1B** presents the time course of the capsaicin effect on the maximal amplitudes of currents (n = 6–8 oocytes). Amplitudes of currents remained unchanged and stable during the course of experiments in the presence of vehicle (0.3% v/v DMSO; n = 5). However, current amplitudes decreased gradually during the application of 100 µM capsaicin and completely recovered after 15 to 20 min of washout period (**Figure 1B**). Inhibition of 5-HT_3_ receptor-induced currents by capsaicin was concentration-dependent with an IC_50_ of 62 ± 5 µM and a slope of 1.4 (**Figure 1C**).

**Figure 2:**
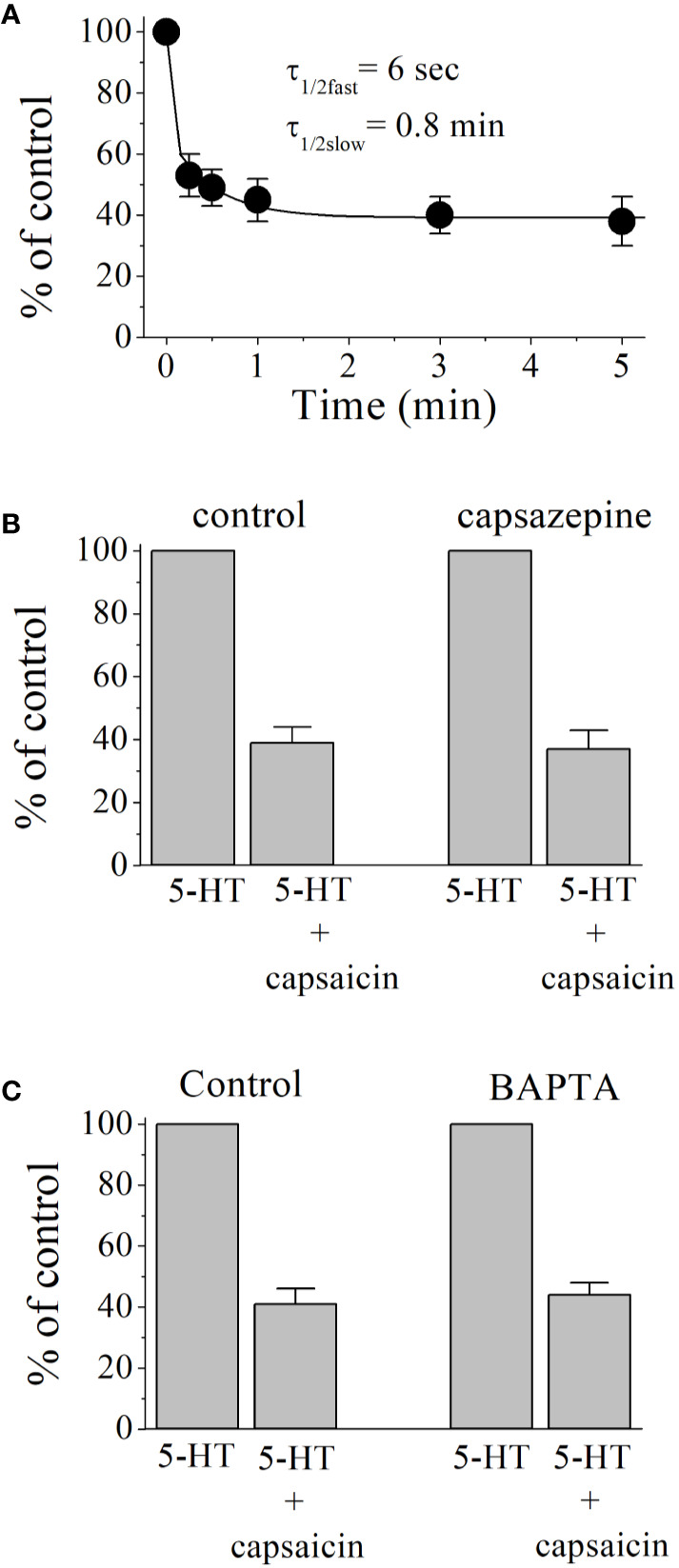
Inhibitory effect of capsaicin on 5-HT_3_ receptor increases with pre-application times and independent of TRPV1 receptors and intracellular Ca^2+^ levels. **(A)** Capsaicin inhibition of 5-HT_3_ receptor as a function of pre-incubation time. Exponential decay curve with two time constants τ_fast_ and τ_slow_, shows the best fit for data point in the figure. Each data point represents the means ± SEM from 7 to 8 oocytes. **(B)** Effects of capsazepine (10 µM) on 5-HT (1 µM) induced currents (n = 5–7). Bars represent the means ± S.E.M. **(C)** Effect of BAPTA injection on the capsaicin inhibition of 5-HT-induced currents. 5-HT (1 μM)-induced currents were recorded before and after 10 min capsaicin (100 μM) application in oocytes injected with 50 nl distilled-water (controls, n = 5) or 50 nl of BAPTA (200 mM, n = 6). Bars represent the means ± S.E.M.

An open-channel blocker would access its binding site during the channel opening time and the extent of drug inhibition would be independent of its pre-incubation time. However, close examination of the time course of capsaicin inhibition showed fast and slow phases with the respective time constants of τ_1/2fast_ = 6 s. and τ_1/2slow_ = 0.8 min, arguing against open channel blockade (**Figure 2A**). Without preincubation, co-application of capsaicin (100 µM) and 5-HT (1 µM) induced a 46 ± 5% inhibition of controls (n = 4). TRPV1 receptors are endogenously expressed and activated by capsaicin in *Xenopus* tropicalis frogs (Ohkita et al., 2012). We have tested the effect of capsazepine (10 µM), a competitive antagonist of TRPV1 receptors (**Figure 2B**) on capsaicin inhibition of 5-HT_3_ receptors. The extent of capsaicin inhibition is not altered in the presence of capsazepine (ANOVA, n = 5–7, P>0.05). Capsazepine (10 µM) alone did not cause any significant change in the amplitudes of 5-HT_3_ receptor-mediated currents (ANOVA, n = 4, P>0.05). Capsaicin has been shown to release Ca^2+^ and interact directly with second messenger pathways (Savitha et al., 1990; Kim et al., 2005; Xu et al., 2012; Chien et al., 2013; Kida et al., 2018). Considering the time course of capsaicin effect, it was possible that capsaicin acts by modulating the effects Ca^2+^ activated kinases on 5-HT_3_ receptor (Zhang et al., 1995; Jones and Yakel, 2003; Hu et al., 2004). Therefore, we tested the effect of the Ca^2+^ chelator BAPTA on capsaicin inhibition of 5-HT_3_ receptors (**Figure 2C**). In oocytes injected with BAPTA, extent of capsaicin (100 µM) inhibition was not significantly different from controls (controls injected with 50 nl distilled water, ANOVA, n = 5–6, P > 0.05), indicating that the effect of capsaicin is not mediated by changes in intracellular Ca^2+^ levels.

**Figure 3:**
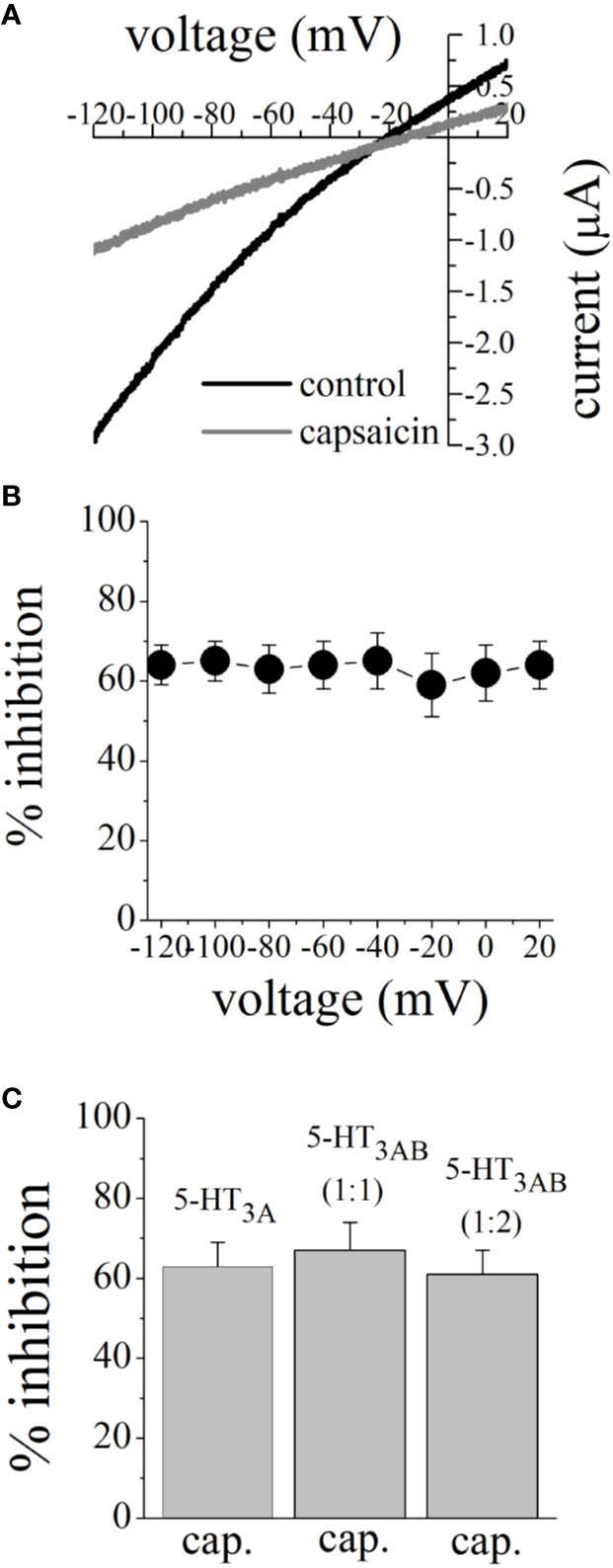
Effects of membrane potential and subunit combination on capsaicin inhibition of 5-HT-activated currents. **(A)** Current-voltage relationships of 5-HT (1 μM)-activated currents before and after 10 min pre-application of 100 µM capsaicin. Data points are the means ± SEM (n = 5) measured from 2-s voltage ramps. **(B)** Inhibition of 5-HT-activated current by 100 µM capsaicin at different membrane potentials. Capsaicin inhibition of 5-HT-activated currents did not change significantly at different membrane potentials (P>0.05, ANOVA; n = 5). **(C)** The effect of 100 μM capsaicin on human 5-HT_3A_, 5-HT_3A_ and 5-HT_3B_ receptors co-expressed in subunit ratios 1:1 and 1:2. Currents were activated by 3 µM and 30 µM 5-HT for 5-HT_3A_ and 5-HT_3AB_ receptor combinations, respectively. The bar graph shows mean ± SEM from 5 to 7 oocytes.

Earlier electrophysiological studies reported that capsaicin inhibits the function of voltage-gated Na^+^ channels (Lundbaek et al., 2005; Wang et al., 2007) and K^+^ channels (Kuenzi and Dale, 1996), and Ca^2+^ channels (Hagenacker et al., 2005) in a voltage-dependent manner. We plotted the current-voltage (I-V) relationships of 5-HT_3_ receptor-induced currents before and after 15 min capsaicin (100 µM) application (**Figure 3A**). Extent of capsaicin inhibition was not altered by changing membrane potentials (**Figure 3B**). Subunit combination of 5-HT_3_ receptors has been shown to alter effects of various drugs (Thompson and Lummis, 2007; Barnes et al., 2009). We compared the effect of capsaicin (100 μM) between 5-HT_3A_ and 5-HT_3AB_ subunits. Results indicated that the extent of capsaicin inhibition was not statistically different among 5-HT_3A_, 5-HT_3AB_ (injected with cRNA ratio of 5-HT_3A_ and 5-HT_3B_ subunits, respectively), and 5-HT_3AB_ (ratio of 1:2) receptors (n = 5–7, ANOVA, P>0.05; **Figure 3C**).

**Figure 4:**
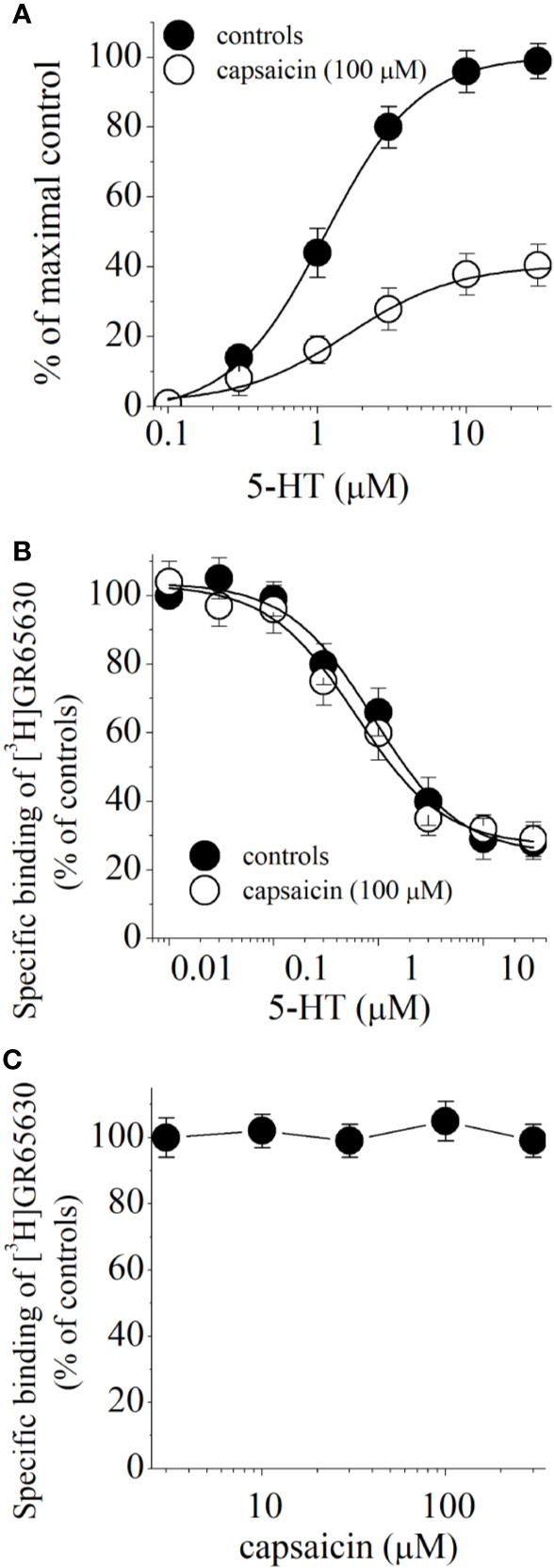
Effect of capsaicin on 5-HT concentration-response relationship and binding of [^3^H]GR65630 to 5-HT_3_ receptor expressed in *Xenopus* oocytes. **(A)** Concentration-response curves for 5-HT-activated currents in the absence and presence of 100 µM capsaicin. Data points represent the mean ± S.E.M. (n = 6–8). The curves depict the best fit of the data to the logistic equation described in the methods. The concentration-response for capsaicin is normalized to maximal control response. **(B)** Effects of capsaicin on the displacement of specific [^3^H]GR65630 binding by nonlabeled 5-HT in oocyte membranes. Membrane preparations were pre-incubated 100 μM capsaicin for 1 hour. The concentration of [^3^H]GR65630 was 1 nM. Data points indicate means ± SEM from 8 to 11 measurements from 3 experiments. **(C)** Effects of increasing concentrations of capsaicin on the specific binding of [^3^H]GR65630 (1 nM). Data points indicate means ± S.E.M from 7 to 10 measurements.

Capsaicin may inhibit 5-HT_3_ receptor by competing with the binding of 5-HT to the receptor. For this reason, we examined 5-HT concentration-responses in the absence and presence of 100 µM capsaicin (**Figure 4A**). Capsaicin inhibited maximal 5-HT responses without causing a significant change in EC_50_ values (in the absence and presence of capsaicin were 1.4 ± 0.3 and 1.9. ± 0.4 μM, respectively; n = 6–8), suggesting noncompetitive inhibition. In radioligand binding experiments, specific binding of [^3^H]GR65630 was inhibited by increasing concentrations of 5-HT in oocyte membranes containing 5-HT_3_ receptor (**Figure 4B**). The IC_50_ values for 5-HT inhibition of [^3^H]GR65630 binding were not significantly altered by 100 μM capsaicin (in the absence and presence of capsaicin were 591 ± 154 and 612 ± 141 nM, respectively; ANOVA, n = 8–11; P>0.05). Similarly, increasing capsaicin concentrations did not change the specific binding of [^3^H]GR65630 (**Figure 4C**).

We also investigated whether the vanillyl group in capsaicin is involved in the inhibition of 5-HT_3_ receptors. Application of vanillin (100 µM, for 15 min), which has only a vanillyl group, did not affect the 5-HT_3_ receptor (ANOVA, n = 8, P>0.05). In contrast, the application of 100 µM dihydrocapsaicin, which contains a vanillyl residue and an acyl chain, inhibited 5-HT_3_ receptors to 62% ± 6% (ANOVA, n = 6–9, P<0.05) suggesting that the inhibition of 5-HT_3_ receptors requires the acyl chain, which causes the compound to be lipophilic.

**Figure 5:**
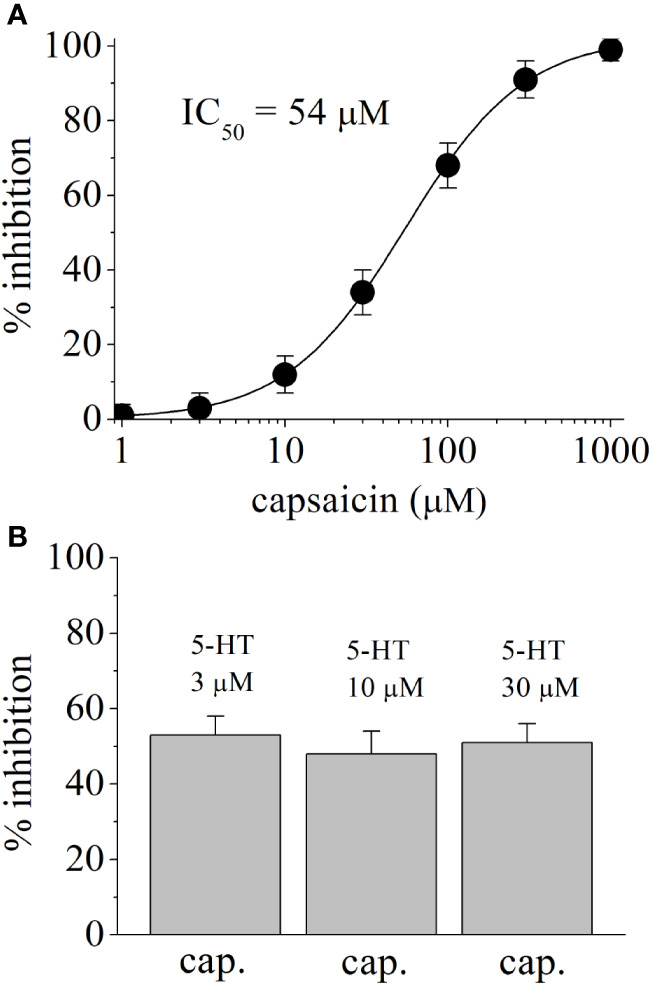
Effects of capsaicin on 5-HT-induced Ca^2+^ influx through human 5-HT_3_ receptors. **(A)** Concentration-dependent inhibition of 5-HT_3_ receptors by capsaicin. Aequorin luminescence induced by 3 μM 5-HT was recorded as a measure of an increased cytosolic Ca^2+^ concentration in coelenterazine *h*-loaded HEK-293-AEQ17 cells heterologously expressing human 5-HT_3_ receptors. Capsaicin was present 10 min before and during 5-HT application. Data are expressed as percentages of the response to 5-HT in the absence of capsaicin (means ± SEM; n = 5). **(B)** The effects of capsaicin (50 µM) on aequorin luminescence activated by 3 μM, 10 µM, and 30 μM 5-HT. Bars represent means ± SEM; n = 16.

In HEK-293-AEQ17 cells transfected with human 5-HT_3_ receptor, application of 5-HT induced concentration-dependent increases in aequorin luminescence with an EC_50_ value of 2.3 μM and slope of 2.7 (n = 4–5 for each concentration point). Aequorin response to 5-HT (10 μM) was completely inhibited by 0.5 μM granisetron (n = 4). In coelenterazine *h*-loaded HEK-293-AEQ17 cells not transfected with 5-HT_3_, injection of 30 μM 5-HT did not cause luminescence activation (n = 4). Application of capsaicin (100 μM) alone did not cause a significant change in baseline aequorin luminescence (n = 4). **Figure 5A** shows the capsaicin inhibition of 5-HT_3_ receptor mediated aequorin responses. Capsaicin inhibited in concentration-dependent manner with an IC_50_ value of 54 μM. **Figure 5B** represents the extent of capsaicin (50 μM) inhibition on aequorin luminescence induced by 3, 10, and 30 μM 5-HT in HEK-293-AEQ cells transfected with human 5-HT_3_ receptor. There was no statistically significant difference in the extent of capsaicin inhibition at increasing 5-HT concentrations (ANOVA, n = 8–11; P>0.05).

**Figure 6:**
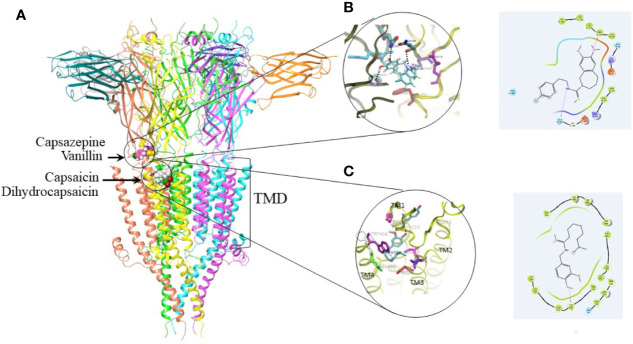
Docking studies on capsaicin, dihydrocapsaicin, capsazepine, and vanillin. **(A)** 5HT_3_ receptor with best ranking poses of the docked capsaicin, dihydrocapsaicin, capsazepine, and vanillin. Proposed binding site for capsazepine and vanillin is located at interface with transmembrane domain (TMD). Proposed binding site for capsaicin and dihydrocapsaicin is located in the TMD. **(B)** 3D and 2D binding interactions within capsazepine binding pocket showing potential key residues. Residues that form hydrogen bond with capsazepine are shown in CPK color. **(C)** 3D and 2D binding interactions within capsaicin binding pocket showing potential key residues. Residue that forms hydrogen bond with capsaicin is shown in CPK color.

The results of docking calculations are presented in **Figure 6**. All binding poses of capsazepine are located at the interface between transmembrane domain (TMD) and extra cellular domain. This binding site is situated in similar position with allosteric binding site predicted by (Lohning et al., 2016). Free energy of binding predicted by Autodock Vina for most favorable docking pose is −7.8 kcal/mol. Gold has predicted similar binding poses for capsazepine. Inside of the capsazepine binding pocket Gln56 and Pro274 form hydrogen bonds with the hydroxyl on the benzazepine group of capsazepine, while Gln184 makes hydrogen bond with amide group of the capsazepine. Phe222 as well as backbone part of the Glu53 and Lys54 interact with the benzazepine moiety of the capsazepine.

The preferable positions of capsaicin and dihydrocapsaicin are located in upper part of the TMD between TM4, TM3 and TM1. Free energy of binding predicted by Vina for best ranking pose of capsaicin is −7.8 kcal/mol. Dihydrocapsaicin has comparable binding free energies. Gold docking program has predicted similar binding poses. Capsaicin alkyl chain makes hydrophobic interaction with the amino acids residues located on TM3 helix such as Ile283, Leu282 as well as with the backbone of Tyr286. This alkyl chain also makes hydrophobic contact with the amino acids residues located on TM4 helix such as Trp454, Trp456, and Leu455. Methoxyphenyl group of capsaicin makes hydrogen bond with the backbone of the Tyr223 located on the TM1 helix. The interaction of the capsaicin that includes three helices belonging to the one subunit of the TMD may interfere with the pore opening that includes rearrangement of transmembrane helices. It can explain experimental funding presented in this paper that capsaicin works as effective negative allosteric modulator. The results of the docking calculations for vanillin show that it can bind to both binding pockets with similar predicted binding free energy −4.8 kcal/mol. In the transmembrane binding pocket, vanillin is situated between TM4 and TM3 helices. Aldehyde group of vanillin interacts with the Tyr286 on TM3 helix, hydroxyl group makes hydrogen bond with Asn141. Phenol makes hydrophobic contact with Trp459 on TM4.

## Discussion

Results indicate that capsaicin inhibits the function of human 5-HT_3_ receptors. Inhibition by capsaicin is time and concentration dependent with IC_50_ values of 62 and 54 µM in *Xenopus* oocytes and HEK-293 cells, respectively. The results of functional and radio-ligand binding studies indicate that capsaicin does not share the same binding site with 5-HT and act as a negative allosteric modulator of 5-HT_3_ receptor.

Capsaicin has been shown to release Ca^2+^ from intracellular stores, modulate store-operated Ca^2+^ channels, and interact with various Ca^2+^ sensitive kinases in a TRPV1 receptor-independent manner (Savitha et al., 1990; Kim et al., 2005; Xu et al., 2012; Chien et al., 2013; Kida et al., 2018). Considering the time-course of capsaicin effect, it was possible that Ca^2+^ activated kinases may be involved. However, capsaicin inhibition of 5-HT_3_ receptor remained unaltered in oocytes injected with BAPTA. Furthermore, capsaicin alone did not cause changes in holding current, which is moderately sensitive to Ca^2+^ due to the presence of Ca^2+^-activated Cl^−^ channels in *Xenopus* oocytes (Dascal, 1987). Similarly, application of capsaicin alone did not activate aequorin luminescence in HEK-293 cells, suggesting that intracellular Ca^2+^ is not involved in observed effects of capsaicin.

Capsaicin in the concentration ranges used in this study has been shown to act directly on various ion channels in a TRPV1 independent manner. Capsaicin directly modulates the functions of voltage-gated Na^+^ channels (Bielefeldt, 2000, IC_50_ = 40 µM; Duan et al., 2007, IC_50_ = 39 µM; Wang et al., 2007, IC_50 =_ 76 µM; Tomohiro et al., 2013, IC_50_ = 100 µM), K^+^ channels (Grissmer et al., 1994, IC_50 =_ 158 µM; Kuenzi and Dale, 1996, IC_50_ = 21 µM; Wu et al., 2011, IC_50 =_ 103 µM; Aréchiga-Figueroa et al., 2017, IC_50_ = 10 µM), and Ca^2+^ channels (Kuenzi and Dale, 1996, IC_50_ = 44 µM; Castillo et al., 2007, IC_50_ = 38 µM). In the present study, capsaicin inhibited 5-HT_3_ receptors with IC_50_ values of 54 and 62 µM which are comparable to values obtained in other studies on the direct effects of capsaicin.

In various dermatological disorders, topical application of capsaicin has been widely used for analgesia and shown to provide adequate absorption from the skin and good bioavailability (Rollyson et al., 2014). In topically applied preparations, the concentration of capsaicin ranges between 3 and 260 mM (0.1–8%) (Bley, 2013). Assuming that 2% of topically applied capsaicin is absorbed into the skin (Lee et al., 1997; Wohlrab et al., 2015), it is likely that the concentration of capsaicin in the dermis ranges between 60 and 5.2 mM for 0.1% and 8% cutaneous applications, respectively. Importantly, membrane concentration of capsaicin is expected to be greatly higher than that in extracellular compartments due to its high lipophilic structure with a LogP (octanol–water partition coefficient) value of 3.8 (Rollyson et al., 2014; Swain and Kumar Mishra, 2015). Following subcutaneous or intravenous administration in animals, the concentrations of capsaicin in the brain and spinal cord were approximately 5-fold higher than that in blood (O’Neill et al., 2012). Thus, modulation of 5-HT_3_ receptors demonstrated in this study may be of pharmacological relevance.

In electrophysiological studies, capsaicin inhibited the maximum 5-HT responses without altering EC_50_ of the 5-HT, indicating that capsaicin does not compete with the 5-HT binding site of the receptor. In addition, in radio-ligand binding studies, binding of competitive 5-HT3 receptor antagonist [^3^H]GR65630 was not significantly affected by capsaicin, further suggesting that capsaicin does not interact with the 5-HT binding site. Furthermore, aequorin luminescence studies in HEK-293-AEQ17 cells indicated that the extent of capsaicin inhibition of aequorin responses was not changed significantly by increasing 5-HT concentrations. Thus, the results of electrophysiological, luminescence, and radioligand binding experiments indicate that capsaicin acts as an allosteric inhibitor of 5-HT_3_ receptor. Importantly, in a recent in silico docking study, a high scoring allosteric and hydrophobic capsaicin binding site located at the interface between the extracellular and transmembrane domain of 5-HT_3A_ receptor subunit has been identified (Lohning et al., 2016). Our results are also in agreement with an earlier study investigating the effects of more than 200 odorous compounds, terpenes, alcohols, and pungent substances (Ziemba et al., 2015), reporting that various gingerol derivatives, capsaicin and polygodial, inhibit 5-HT_3_ receptors. Furthermore, our results indicated that dihydrocapsaicin, but not vanillin, inhibited 5-HT_3_ receptor, suggesting that the lipophilicity is an important property for capsaicin effect on this receptor.

As a highly lipophilic agent, capsaicin has been shown to alter physicochemical properties of cell membranes, perturb the bilayer structure, and inhibit the functions of various ion channels (Lundbaek et al., 2005; Lundbaek et al., 2010; Ingólfsson et al., 2014). Thus, it is likely that capsaicin first dissolves into the lipid membrane, changes the physicochemical properties of the cell membrane and, subsequently or simultaneously, diffuses and reaches to binding site(s) located on the transmembrane domains of the 5-HT_3_ receptor. Consistent with this assumption, direct effects of capsaicin on several ion channels including the 5-HT_3_ receptor usually require several minutes to reach steady-state maximal levels. Similarly, several minutes of application times (5–15 min) are prerequisite for actions of several lipophilic and allosteric modulators such as steroids, endocannabinoids, and cannabinoids (Oz et al., 2002a; Oz et al., 2002b; Yang et al., 2010a; Yang et al., 2010b) on 5-HT_3_ receptors (for reviews, Oz, 2006; Oz et al., 2015; Al Kury et al., 2018), suggesting that the binding site(s) for these allosteric modulators is located inside the lipid membrane. Notably, these results also indicate that drug exposure time rather than channel opening is important for the effects of these lipophilic modulators, suggesting that they can interact with the channel during the closed state.

Computational results suggest that capsaicin and dihydrocapsaicin bind to allosteric transmembrane binding site situated between transmembrane (TM), TM1, TM2, TM3, and TM4 in close proximity to extracellular domain. Capsaicin and dihydrocapsaicin make hydrophobic interactions with TM4, TM3 and hydrogen bond with TM1, which may stabilize 5HT_3_ in closed conformation. Capsaicin and dihydrocapsaicin have bended conformation inside of the binding pocket where flexible alkyl tail is situated between TM4 and TM3 making hydrophobic contact with them. According to the docking calculations, capsazepine has preferable binding position between extracellular and transmembrane domain making hydrogen bonds inside of the binding site. Although capsazepine is structural analog of capsaicin it is less flexible.Vanillin binds to both allosteric binding sites with similar probability while in the transmembrane binding site it makes interactions with the amino acids located on TM3 and TM4 helices.

Recently, capsaicin has been shown to inhibit glycine (Thakre and Bellingham, 2017; Thakre and Bellingham, 2019) and α7-nicotinic acetylcholine receptors (Alzaabi et al., 2019) indicating that, in addition to 5-HT_3_ receptors, other members of ligand-gated ion channel family are also targets mediating wide range of pharmacological actions of capsaicin. In conclusion, our results indicate that capsaicin acts as a negative allosteric modulator of not only homomerically, but also heteromerically (5-HT_3AB_ with 1:1 and 1:2 ratio) expressed human 5-HT_3_ receptor.

## Data Availability Statement

The raw data supporting the conclusions of this article will be made available by the authors, without undue reservation, to any qualified researcher.

## Ethics Statement

The animal study was reviewed and approved by the Institutional Animal Care and Use Committee at the College of Medicine and Health Sciences, United Arab Emirates University (Protocol A9/08).

## Author Contributions

EEN, TP, LH, and AHA conducted experiments and analyzed the data. TP, DEL, K-HSY, and FCH assisted on data analysis and writing the manuscript. MO planned and organized the study. All authors contributed to the article and approved the submitted version.

## Funding

The research in this study was supported by grants from CMHS, UAE University and Kuwait University-The Kuwait Foundation for the Advancement of Sciences (KFAS).

## Conflict of Interest

The authors declare that the research was conducted in the absence of any commercial or financial relationships that could be construed as a potential conflict of interest.
